# Identification of DNA-binding proteins using support vector machines and evolutionary profiles

**DOI:** 10.1186/1471-2105-8-463

**Published:** 2007-11-27

**Authors:** Manish Kumar, Michael M Gromiha, Gajendra PS Raghava

**Affiliations:** 1 Bioinformatics Centre, Institute of Microbial Technology, Sector 39A, Chandigarh-160036, India; 2 Computational Biology Research Center, National Institute of Advanced Industrial Science and Technology, AIST Tokyo Waterfront Bio-IT Research Building, 2-42 Aomi, Koto-ku, Tokyo 135-0064, Japan

## Abstract

**Background:**

Identification of DNA-binding proteins is one of the major challenges in the field of genome annotation, as these proteins play a crucial role in gene-regulation. In this paper, we developed various SVM modules for predicting DNA-binding domains and proteins. All models were trained and tested on multiple datasets of non-redundant proteins.

**Results:**

SVM models have been developed on DNAaset, which consists of 1153 DNA-binding and equal number of non DNA-binding proteins, and achieved the maximum accuracy of 72.42% and 71.59% using amino acid and dipeptide compositions, respectively. The performance of SVM model improved from 72.42% to 74.22%, when evolutionary information in form of PSSM profiles was used as input instead of amino acid composition. In addition, SVM models have been developed on DNAset, which consists of 146 DNA-binding and 250 non-binding chains/domains, and achieved the maximum accuracy of 79.80% and 86.62% using amino acid composition and PSSM profiles. The SVM models developed in this study perform better than existing methods on a blind dataset.

**Conclusion:**

A highly accurate method has been developed for predicting DNA-binding proteins using SVM and PSSM profiles. This is the first study in which evolutionary information in form of PSSM profiles has been used successfully for predicting DNA-binding proteins. A web-server DNAbinder has been developed for identifying DNA-binding proteins and domains from query amino acid sequences .

## Background

DNA-binding proteins (DNA-BPs) are very important constituent of both eukaryotic and prokaryotic proteomes. It has been reported that approximately 2–3% of prokaryotic and 6–7% of eukaryotic proteins can bind to DNA [1,2]. These proteins play important roles in DNA packaging, replication, transcription regulation and other activities associated with DNA. Hence proteins that target specific DNA sequences can be a potential therapeutics for genetic diseases and cancers. In the form of restriction enzymes, DNA-BPs play a crucial role in prokaryotic host defence. Due to different functions DNA-BPs are diverse group of proteins both in terms of amino acid sequences and three-dimensional structures. Hence, identification of DNA-BPs can play a vital role in proteome annotation and understanding an important class of proteins.

In past, several methods have been developed for predicting DNA-BPs. Broadly, these methods can be divided into two categories i) prediction from protein structure and ii) prediction from amino acid sequence. Structure based prediction methods discriminate DNA-binding and non-binding proteins with high accuracy on the basis of positively charged electrostatic patches [2], DNA-binding structural motifs [3], protein sequence composition, solvent accessibility and secondary structure [4], net charge, dipole and quadrapole moments, [5] and size of largest positive surface patch and amino acid composition [6]. Unfortunately, these methods can't be used in high throughput annotation, as they require the structure of a protein for prediction. Cai and Lin 2003, used pseudo-amino acid composition as input for support vector machine (SVM) to discriminate RNA, rRNA and DNA binding proteins from non-binding proteins (NBPs) [7]. Recently Yu et al. (2006) [8] has developed a SVM based method for prediction of rRNA, RNA and DNA binding proteins. They used a feature vector of dimension 132, which includes amino acid composition and composition of physico-chemical properties.

In this work, a systematic attempt has been made to predict DNA-BPs from their amino acid sequences using various features of proteins, like amino acid composition. First, we analyzed the amino acid composition of DNA-binding proteins and based on the observation, SVM models have been developed using amino acid, dipeptide and four-part amino acid compositions of proteins. Besides composition, we also developed SVM models using PSSM profiles obtained from PSI-BLAST. We also examined the performance of similarity search (BLAST and PSI-BLAST) and motif-finding (MEME/MAST) approaches. All models developed in this study were evaluated using five-fold cross validation technique.

## Results

### SVM models

#### Prediction of DNA-binding domains/chains

**Figure 1:**
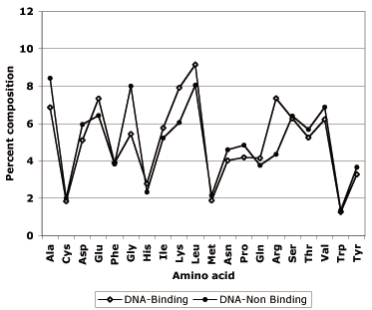
Percentage composition of DNA-binding and non-binding proteins in main dataset (DNAset).

**Figure 2:**
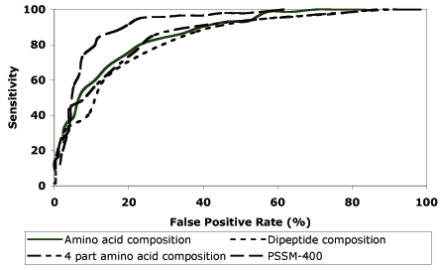
Performance of SVM models on DNAset dataset (146 DNA-binding and 250 non-binding proteins) in the form of ROC plot.

SVM models have been developed on DNAset or main dataset, which has DNA-binding and non-binding chains obtained from PDB. First, composition based SVM model has been developed for predicting DNA-binding domains and achieved the accuracy of 79.80% with MCC, 0.58 (Table 1). In order to understand the high success rate, we compared the amino acid compositions of DNA-binding and non-binding domains in DNAset (Figure 1). As shown in Figure 1, few residues like Lys, Arg and Glu are abundant in DNA-binding domains where as other residues like Gly are less frequent in DNA-binding domains. Due to these significant compositional differences DNA-binding domains can be predicted with high accuracy. In general, SVM models based on dipeptides perform better than amino acid composition based models. However, in this study its performance was poor. In addition, earlier studies showed that the performance of split-amino acid composition was better than amino acid composition. Hence, we developed SVM models using amino acid composition of four non-overlapping parts of a protein. As shown in Table 1, SVM model developed with four-part composition was not as efficient as amino acid composition based model. Further, it has been well documented that evolutionary information in form of PSSM profiles provides more information, which significantly improved the accuracy of prediction in several studies, such as RNA binding sites, subcellular localization, β-turns etc [9-13]. Thus, we developed SVM models using PSSM profiles and achieved the overall accuracy of 86.62% with MCC, 0.72. The performance of all SVM modules developed using DNAset is shown in form of ROC plot in Figure 2. We also performed self-consistency test and achieved very high accuracy (See Additional file 1, Table S1).

**Table 1:** The performance of SVM models developed using different types of compositions. These models were trained and tested on DNAset, a dataset of DNA-binding and non-binding protein domains/chains.

**Composition Type**	**Threshold**	**Sensitivity**	**Specificity**	**Accuracy**	**MCC**
**Amino Acids**	0.3	78.11	80.80	79.80	0.58
**Dipeptides**	0.0	73.35	77.60	76.01	0.50
**4-parts amino acids**	0.0	77.45	77.60	77.53	0.54
**PSSM**	0.1	86.32	86.80	86.62	0.72

#### Prediction on DNA-binding proteins

We developed SVM models on DNAaset, which consists of 1153 DNA-binding proteins and equal number of non-binding proteins. This dataset have full-length proteins extracted from Swiss-Prot. As shown in Table 2, with amino acid composition we achieved the accuracy of 72.42% with MCC, 0.45. The performance of SVM model improved significantly using evolutionary information obtained from PSSM profiles, which raised the accuracy and MCC to 74.22% and 0.49, respectively. Performance of all SVM modules in form of ROC plot is shown in Figure S1.

**Table 2:** The performance of SVM models developed on DNAaset and evaluated using five-fold cross-validation technique.

**Composition Type**	**Threshold**	**Sensitivity**	**Specificity**	**Accuracy**	**MCC**
**Amino Acids**	0.2	72.51	72.33	72.42	0.45
**Dipeptides**	0.1	72.59	70.59	71.59	0.43
**4-parts amino acids**	0.0	70.85	70.24	70.55	0.41
**PSSM**	-0.3	73.53	74.92	74.22	0.49

#### Quality of PSSM profiles

As shown in Table 1, the PSSM based SVM models perform better than other models. In order to examine the effect of PSSM quality on the performance of SVM model, we generated PSSM profiles from our training dataset (4/5 part of DNAset) instead of 'nr' protein database. The performance of SVM model using PSSM decreased significantly from 86.62% to 79.54% when PSSM generated from DNAset instead of "nr" (See Additional file 1, Table S2). We examined the reason for the poor performance and found that a large number of query protein did not have any PSI-BLAST hit when searched against DNAset. Thus, we evaluated performance of SVM model on proteins having PSI-BLAST hits (24 DNA-BPs and 56 non-binding proteins at e-value 0.1) and proteins having no PSI-BLAST hits (122 DNA-BPs and 194 non-binding proteins). We achieved the accuracy of 83.75% for proteins having PSI-BLAST hits and accuracy of 78.48% for proteins having no PSI-BLAST hits (Table 3). The PSSM based SVM model performed worse than SVM model based on amino acid composition on proteins having no PSI-BLAST hit. The SVM model based on PSSM generated from "nr" database performed better than that generated with our dataset (even if there were PSI-BLAST hits). This demonstrates that performance of PSSM based SVM model is affected by quality of PSSM and performs better if PSSM is generated from similar sequences. In the absence of similarity its performance will be poor (See Figure S2).

**Table 3:** The performance of PSSM based SVM models on proteins with and without PSI-BLAST hits at e-value 0.1 against DNAset. These models were trained and tested on DNAset.

**Threshold**	**With PSI-BLAST hit**		**Without PSI-BLAST hit**	
	
	**Sensitivity**	**Specificity**	**Sensitivity**	**Specificity**
-1	95.83	48.21	97.54	42.78
-0.9	95.83	53.57	96.72	44.33
-0.8	95.83	53.57	96.72	45.88
-0.7	95.83	57.14	95.90	48.97
-0.6	91.67	62.50	95.90	51.55
-0.5	91.67	64.29	95.08	54.12
-0.4	91.67	66.07	93.44	57.73
-0.3	87.50	66.07	92.62	60.31
-0.2	83.33	69.64	91.80	63.40
-0.1	83.33	73.21	89.34	66.49
0	83.33	76.79	86.89	69.07
0.1	83.33	82.14	83.61	71.65
0.2	83.33	83.93	80.33	75.77
**0.3**	**83.33**	**83.93**	**77.87**	**78.87**
0.4	75.00	85.71	73.77	81.44
0.5	75.00	85.71	69.67	82.99
0.6	75.00	89.29	64.75	85.05
0.7	75.00	92.86	60.66	88.66
0.8	62.50	94.64	56.56	89.69
0.9	62.50	96.43	50.00	91.75
1	54.17	96.43	45.08	93.30

#### Performance on blind dataset

It is important to examine the performance of the newly developed model on an independent dataset. In this study, we evaluated the performance of our SVM models (trained on DNAset) on independent dataset called DNAiset, which consists of 100 NBPs (BindN_testsp) and 92 DNA-BPs (BindN_testpdb) obtained from Wang and Brown (2006) [14]. At default threshold of 0.1, our PSSM based SVM model correctly predicted 70 out of 92 DNA-BPs and 89 out of 100 NBPs (See Additional file 1, Table S3). This demonstrates that our SVM model performs equally well on independent dataset.

#### Performance on realistic dataset

In real life, the number of non-binding proteins is significantly higher than DNA-BPs. Thus, it is important to build and evaluate SVM models on more realistic data rather than equal number of DNA-BPs and NBPs. Hence, we developed a realistic dataset (DNArset), which has 146 DNA-binding domains and 1500 NBPs. First, we developed SVM model using amino acid composition on DNArset and achieved the maximum MCC of 0.40 with accuracy, 89.31%. Then we developed PSSM based SVM model and achieved the maximum MCC of 0.57 with accuracy, 92.59%. As shown in Table 4, PSSM based model performed better than composition-based model both in terms of sensitivity and specificity. These results further confirmed the importance of evolutionary information in predicting the DNA-BPs.

**Table 4:** The Performance of SVM models using amino acid and PSSM profiles on a realistic dataset (DNArset).

**Threshold**	**Amino acid composition based model**				**PSSM based model**			
	
	Sn*	**Sp**	**Acc**	**MCC**	**Sn**	**Sp**	**Acc**	**MCC**
-1.00	80.21	63.67	65.13	0.26	91.75	77.47	78.73	0.44
-0.90	77.47	67.00	67.92	0.26	89.70	78.60	79.58	0.44
-0.80	76.78	70.67	71.20	0.29	87.66	80.20	80.86	0.45
-0.70	74.74	74.40	74.43	0.31	85.61	81.60	81.95	0.45
-0.60	74.05	77.07	76.79	0.33	84.23	83.20	83.29	0.46
-0.50	71.98	79.27	78.62	0.34	78.73	84.20	83.72	0.44
-0.40	69.91	81.13	80.13	0.34	78.05	85.67	84.99	0.46
-0.30	69.22	83.60	82.32	0.37	74.60	87.27	86.15	0.46
-0.20	65.79	85.73	83.96	0.38	73.22	89.07	87.66	0.48
-0.10	60.99	87.40	85.06	0.37	71.15	91.00	89.24	0.51
0.00	58.23	89.13	86.39	0.38	70.46	92.27	90.34	0.53
0.10	53.40	90.40	87.12	0.37	68.41	93.60	91.37	0.55
0.20	50.02	92.20	88.46	0.38	65.68	94.60	92.04	0.56
**0.30**	**47.95**	**93.33**	**89.31**	**0.40**	**63.61**	**95.40**	**92.59**	**0.57**
0.40	44.53	93.93	89.55	0.38	60.16	95.87	92.71	0.56
0.50	41.79	94.60	89.91	0.38	53.31	96.60	92.77	0.53
0.60	37.70	95.13	90.04	0.35	51.29	97.07	93.01	0.54
0.70	32.90	95.73	90.16	0.33	47.86	97.53	93.13	0.52
0.80	30.83	96.40	90.58	0.33	42.39	97.87	92.95	0.49
0.90	30.16	97.00	91.07	0.35	38.30	98.40	93.07	0.48
1.00	28.78	97.33	91.25	0.35	34.87	98.67	93.01	0.47

* **Sn: **Sensitivity; **Sp: **Specificity; **Acc: **Accuracy

### Comparison with existing methods

It is important to compare the performance of newly developed method with existing methods in order to demonstrate its capabilities. We compared the performance of above SVM models with similarity and motif-based approaches, which are commonly used for functional annotation of proteomes. As shown in Additional file 1, Table S4, the sensitivity of both modules DNA-BLAST [15] and DNA-PSIBLAST [16] was about 10% at e-value of 0.1, when evaluated on DNAset using five fold cross-validation technique. We also evaluated the performance of MEME/MAST on DNAset and observed poor performance, where only 15 domains showed motif at e-value cut-off of 1. These results demonstrated that our SVM models perform better than commonly used techniques like BLAST, MEME/MAST.

Most of the existing methods predict DNA binding proteins from proteins structures. These structure based prediction methods are not suitable for high throughput genome annotation, as they require the structure of a protein. Best of authors knowledge, three methods have been developed in the past for predicting DNA-BPs from their amino acid sequences. Ahmad et al. (2004) [4] developed a neural network based method using amino acid composition and reported the accuracy of 64.5%. They used small but clean and non-redundant dataset, which consists of 62 DNA-BPs obtained from PDB and 915 non-binding proteins obtained from Swiss-Prot. Their dataset was similar to our main dataset (DNAset), where we achieved the maximum accuracy of 79.80% using amino acid composition and 86.62% using PSSM profiles. Cai and Lin (2003) [7] developed a SVM based method using pseudo-amino acid composition and obtained the average accuracy of 81%. Although they compiled a large dataset, it was neither non-redundant nor clean because it contained all proteins including probable DNA-BPs. Yu et al. (2006) [8] developed a SVM based method using various physical-chemical properties and showed an average accuracy of 71.64%. They collected binding and non-binding proteins as proposed by Cai and Lin (2003) [7] from Swiss-Prot and created a non-redundant dataset of 1153 DNA-BPs and 1153 non-binding proteins. On alternate dataset (DNAaset) which was identical to Yu et. al. (2006) [8], we achieved an accuracy of 74.22% using PSSM based SVM model. This demonstrates that our method performs better than other existing methods. The usage of SVM, which performs better than ANN, particularly on small dataset and evolutionary information in the form of PSSM profiles, improved the performance of the present method.

### Prediction of DNA-binding domains *vs *proteins

In this study, we developed modules for predicting DNA binding proteins using two types of datasets; i) DNAset consists of partial sequences (binding regions) or DNA binding domains, and ii) DNAaset consists of full-length DNA-binding proteins. The question arises whether modules trained on DNAset (domains or partial sequences) will be applicable for predicting full DNA-binding protein or vice versa. We predicted the proteins in DNAaset using amino acid composition based SVM module trained on DNAset and achieved the accuracy of about 55%. Similarly, we predicted proteins in DNAset using amino acid composition based SVM module trained on DNAaset and achieved the accuracy of about 63%. This revealed that the model trained on partial sequences or domains is not valid for predicting full DNA binding proteins and vice versa. In order to understand the reason of failure, we computed and compared amino acid compositions of DNA binding and non-binding proteins in DNAaset (Figure S3). The DNA-binding and non DNA-binding proteins have significantly different amino acid compositions in DNAset whereas such a trend is not observed in DNAaset (Figure 1 & S3). It is due to the fact that DNAaset has full length DNA-binding proteins, which may also have other domains including non DNA-binding domains. Thus, prediction performance of methods trained and tested on DNAset are more accurate than on DNAaset. It also explains the reasons for the failure of methods trained on full proteins (DNAaset) and tested with domain dataset (DNAset), and vice versa. This shows that separate methods are necessary for predicting DNA-binding domains and DNA-binding proteins.

It is also possible that our models were over trained. In order to rule-out this possibility, we evaluated the performance with other existing methods. We found two methods available for public: (i) DBS-PRED developed on DNA-binding domains [4] and (ii) SVM-Prot developed on full DNA-binding proteins [17]. The performance of DBS-PRED was evaluated on a dataset of 100 DNA binding and 100 non-binding proteins extracted from Swiss-Prot. As shown in Additional file 1, Table S5, we achieved the maximum performance of 63% at 30% probability threshold. This means that the performance on DBS-PRED is also poor on full proteins. Similarly, the performance of SVM-Prot was evaluated on DNAset; SVM-Prot predicted 49 out of 146 DNA-binding proteins and 203 out of 250 non-binding proteins when all hits are considered for evaluation; if only top hit was considered SVM-Prot was able to identify13 out of 146 sequences (DNA binding domains/regions) as DNA-binding proteins. This analysis showed that SVM-Prot developed on full protein is not suitable for partial or domain sequences.

## Webserver

The prediction method described in this paper is implemented in the form of a web-server DNAbinder (for detail descriptions please see section Availability and Requirements). The common gateway interface script of DNAbinder is written using PERL version 5.03. DNAbinder server is installed on a Sun Server (420E) under UNIX (Solaris 7) environment. This server allows users to predict DNA-binding proteins using amino acid composition and PSSM based SVM models trained on DNAset, DNArset and DNAaset. Models trained on DNAset or DNArset are suitable for predicting DNA-binding domains/chains, whereas models trained on DNAset are suitable for predicting DNA-binding full length proteins. Server allows submission of multiple sequences for prediction in case of composition based model and submission of one sequence at a time in case of PSSM based model. In case of PSSM based model, we use this model if PSI-BLAST finds significant hits for query sequence; otherwise only simple composition based model is used for prediction.

## Discussion

DNA-binding proteins are one of the major classes of proteins playing a central role in cellular metabolism. Due to their importance in the regulation of gene-expression and other processes, several methods have been developed for predicting DNA-BPs. Most of them predict DNA-BPs using their structural information. These methods have limited scope because the structures are unknown for most of the proteins. In this paper, we developed a highly accurate method for predicting DNA-BPs from their amino acid sequences. The reliability of any prediction method mainly depends on clean and valid dataset. In past, two types of datasets have been used: (i) small and clean dataset with experimentally validated DNA-BPs obtained from PDB [4] and (ii) large dataset, where sequences were obtained from Swiss-Prot [7,8]. In this study we developed our models on two datasets called DNAset or main dataset and DNAaset or alternate dataset in order to benchmark our newly developed method with existing methods. Most of our study was based on DNAset because it has clean and experimentally validated DNA-BPs where as DNAaset may also include fragments and putative proteins.

In any functional annotation, the primary step is to search a query protein against database of annotated proteins (e.g. Swiss-Prot) and assign the function if query protein has significant similarity with target proteins. Similarity based annotation is highly accurate if an experimentally annotated homologous protein is found. But the major challenge is to predict function of a protein in absence of significant similarity. Thus, we developed a method using non-redundant dataset, where similarity between proteins in test and training datasets is very low. We applied similarity search and motif finding techniques on our dataset and found poor performance, as expected. Hence, we have developed ANN and SVM models on DNAset using amino acid and dipeptide compositions for predicting DNA-BPs. We achieved an accuracy of 68.46% from ANN model using amino acid composition, which is slightly better than Ahmad et al., 2004 [4] (See Additional file 1, Table S6). It may be due to the increase in the number of DNA-BPs in DNAset. It is interesting to note that the performance of SVM was significantly better than ANN. Thus, we developed rest of the models using SVM. We achieved significantly high accuracy of 79.80% using SVM model based on simple amino acid composition. This demonstrates the importance of machine learning techniques in prediction of DNA-BPs. In order to enhance the performance further, SVM models have been developed using dipeptide and four-part compositions. Unexpectedly, the performance of dipeptides and four-part composition based SVM models is poorer than composition-based SVM model. The standard techniques like BLAST, PSI-BLAST and MEME/MAST failed to predict DNA-BPs when tested on DNAset due to low similarity among domains/chains in DNAset.

This is the first study that used evolutionary information to discriminate DNA-BPs from non-binding proteins. We extracted evolutionary information of a protein from PSSM profiles obtained form PSI-BLAST search against "nr". The accuracy of the method improved significantly from 79.80% to 86.62% using PSSM profiles, when evaluated on DNAset. The quality of PSSM profiles, of a query sequence depends on similar sequences in a target database. As shown in Table 3, performance of SVM based model decreases significantly when PSSM generated from DNAset instead of "nr" dataset. This is due to fact that "nr" is a very large database in comparison to DNAset and hence the chances of getting similar sequences are very high when a sequence is searched against "nr" database. Thus, quality of PSSM profiles will be superior in case of "nr". We also examined PSI-BLAST hits and observed that each protein in DNAset has BLAST hits when searched against "nr" database. We also demonstrate that PSSM based models perform better, if there is significant BLAST hits (Table 3). As database of "nr" is growing exponentially due to number of sequencing projects the quality of PSSM profiles will improve over the times, which eventually improve the performance of PSSM based models. In DNAbinder server, first we examine whether a query sequence has any BLAST hit in "nr"; if yes, PSSM based model will be used and otherwise amino acid composition based SVM model will be used for prediction.

It is well known that similarity/motif based methods perform better than *ab-initio *classifiers if a query protein has significant similarity with target proteins. The major challenge is to develop a method, which can identify a novel DNA binding protein even if it has no sequence similarity with any of the known DNA-BPs. Hence, we took non-redundant proteins in our main dataset. If we have taken redundant proteins then similarity based method would have performed better than other methods. Our SVM model based on amino acid composition performed well on a dataset where similarity based method failed to detect DNA-BPs. This demonstrates that models developed in this study are capable to identify novel DNA-BPs. The SVM model based on PSSM further improved the accuracy by 6–7%. The question arises why we generate PSSM from 'nr' protein database instead of our own dataset whereas we are assessing BLAST/PSI-BLAST and MEME/MAST on DNAset. There is a fundamental difference between BLAST/PSI-BLAST searching and SVM model using PSSM profiles. In case of BLAST/PSI-BLAST one need to search only against well annotated proteins because we have to assign the same function to query protein if it has similarity with target protein. In case of PSSM it is not important to search a query sequence against the annotated proteins only because we do not assign function based on similarity. It is not important whether query sequence has similarity with DNA-BPs, NBPs or un-annotated proteins for generating PSSM profiles. Thus, one may create PSSM from any database like "nr" in contrast to similarity-based methods, where we need to search against well-annotated proteins only.

The main objective of this study is to develop a prediction method for identification of new DNA-binding proteins and particularly annotating the newly sequenced genomes. Most of the existing methods developed in past have been trained on experimentally annotated DNA-binding protein chains/domains obtained from PDB. The models developed on dataset of protein chains like DNAset are suitable for predicting DNA binding domains but not for predicting DNA-BPs. In order to develop method for predicting DNA-binding proteins, we also developed SVM model using DNAaset, consists of full-length DNA-binding and non-binding proteins. Thus, models developed on DNAaset will be suitable for predicting DNA-BPs. The performance of our SVM model was slightly better than SVM model of Yu et al, 2006 [8], using amino acid composition on same dataset. The difference in the performance of SVM may be due to optimization of learning parameters as Yu et al., 2006 [8] used the default parameters of SVM_light where as we used optimized parameters. Our SVM model using PSSM profiles performs better than the SVM model of Yu et al. 2006 [8], based on physicochemical properties by 3%. These results suggest that evolutionary information is important for predicting DNA-binding proteins.

## Conclusion

We developed a highly accurate method for predicting DNA-BPs using the machine learning technique, SVM. For the first time, evolutionary information has been used to predict DNA-binding proteins. It has been observed that PSSM based models perform better than any other models by 3–7% on all the datasets including independent and realistic datasets. The SVM models developed in this study perform better than other existing methods. One of the major features of this study is that we developed a publicly available web server and stand-alone software, which allows users to identify the DNA-BPs in their dataset of proteins. It was observed that models trained on DNA domains or partial sequences are not suitable for predicting DNA binding proteins and vice versa. Our server, DNAbinder allows users to identify DNA binding domains using the model trained on DNAset and prediction of DNA binding proteins using the model trained on DNAaset. We hope this study will assist the biologist in annotation of genomes.

## Methods

### Datasets

In this study, we used the following datasets to develop various models for predicting DNA-BPs and for evaluating SVM models.

#### DNAset

We extracted 2435 DNA-BPs from Protein Data Bank (PDB) [18] using the keywords, "Protein-DNA complex", "DNA binding" and "DNA binding proteins". All proteins having no DNA chain or having high similarity with other proteins were filtered. Finally, we got 146 non-redundant DNA-BPs in which no two proteins have the sequence identity of more than 25%. A non-redundant set of 250 non-binding proteins was obtained from Stawiski et al. (2003) [2]. They used following criteria: i) no two protein chains have similarity more than 25% and (ii) the approximate size and electrostatics are similar to DNA-BPs. Final dataset called DNAset or main dataset or domain dataset, consists of 146 DNA-binding and 250 non-binding protein chains or domains. We called proteins chains as domains for our convenience, in order to discriminate these PDB chains from full-length DNA-binding proteins obtained from Swiss-Prot.

#### DNAaset

In addition to main dataset (DNAset), we also created an alternate dataset called DNAaset. This dataset consists of 1153 DNA-BPs and 1153 NBPs extracted from Yu et al (2006) [8]. The parent dataset have 88 rRNA-BPs, 377 RNA-BPs, 1153 DNA-BPs and 17779 non-binding proteins. We randomly picked 1153 NBPs and all 1153 DNA-BPs to constitute the alternate dataset (DNAaset). This is non-redundant dataset where no two proteins have more than 25% similarity.

#### DNAiset

In order to evaluate performance of our models on dataset not used for training or testing, we created an independent dataset called DNAiset. This dataset has 92 DNA-binding protein chains obtained from PDB and 100 non-binding proteins picked from Swiss-Prot. These proteins were obtained from BindN server [14].

#### DNArset

Equal number of negative and positives examples is important for developing an efficient classifier. They are also important for evaluating any prediction model where one can simply calculate accuracy for measuring performance. All above datasets have nearly equal number of DNA-binding and non-binding proteins. However, in real life DNA-BPs are significantly less than non-binding proteins. This raises question whether models developed on equal numbers will be effective in real life. Thus, we created a more realistic dataset called DNArset. This dataset has 146 DNA-BPs used in DNAset and 1500 NBPs. These 1500 NBPs were extracted from 17779 non-binding proteins used by Yu et al., 2006 [8] after removing the proteins, whose DNA binding property is not experimentally validated.

### Evaluation of models

We have adopted five fold cross-validation approach to evaluate the performance of all models developed in this study. In this procedure, the whole dataset is randomly divided into five equal parts. Four sets are used for training and remaining one for testing. The procedure is repeated five times in such a way that each set is tested once. This type of sub-sampling test (e.g., 5 or 7-fold cross-validation) is often used to validate the prediction performance of statistical methods. On the other hand, jack-knife test is deemed the most rigorous and objective as analyzed by a comprehensive review [19] and has been increasingly adopted by investigators to test the power of various prediction methods (see, e.g., [20-28]). In order to assess the performance of a model, we computed different parameters: sensitivity, specificity, accuracy and Matthews correlation coefficient (MCC) [29].

### DNA-BLAST and DNA-PSI-BLAST search

In order to assess the performance of similarity search approaches, we evaluated the performance of BLAST [15] and PSI-BLAST [16] on DNAset. We searched proteins of test set against training set proteins using BLAST/PSI-BLAST and assigned a query protein as DNA-binding or non-binding if the first hit was a DNA-BPs or NBPs respectively. We assigned a protein "unknown" if it has no significant similarity with any target protein.

### Support vector machine (SVM)

In this study SVM_light, a freely available software package, has been used to implement SVM. The SVM is a supervised machine-learning method based on the structural risk minimization principle from statistical learning theory. It takes a set of feature vectors as input, along with their output, which is used for training of model. After training, learned model can be used for prediction of unknown examples [30,31]. Detailed description of SVM can be found at Vapnik (1995) [32]. In this work, the SVM training has been carried out by the optimization of various kernel function parameters and the value of the regularization parameter C.

### Protein features and vector encoding

#### Amino acid and dipeptide compositions

The aim of calculating composition of proteins is to transform the variable length of protein sequence to fixed length feature vectors. This is an important and most crucial step during classification of proteins using machine-learning techniques because they require fixed length pattern. The conversion of protein sequence to a vector of 20 dimensions using amino acid composition will encapsulate the properties of a protein into it. In addition to amino acid composition, dipeptide composition was also used for classification that gave a fixed pattern length of 400. The advantage of dipeptide composition over amino acid composition is that it encapsulates information about the fraction of amino acids as well as their local order. The amino acid as well as dipeptide composition was calculated as described below.

comp(i)=RiN×100
 MathType@MTEF@5@5@+=feaafiart1ev1aaatCvAUfKttLearuWrP9MDH5MBPbIqV92AaeXatLxBI9gBaebbnrfifHhDYfgasaacPC6xNi=xI8qiVKYPFjYdHaVhbbf9v8qqaqFr0xc9vqFj0dXdbba91qpepeI8k8fiI+fsY=rqGqVepae9pg0db9vqaiVgFr0xfr=xfr=xc9adbaqaaeGacaGaaiaabeqaaeqabiWaaaGcbaGaem4yamMaem4Ba8MaemyBa0MaemiCaaNaeiikaGIaemyAaKMaeiykaKIaeyypa0tcfa4aaSaaaeaacqWGsbGudaWgaaqaaiabdMgaPbqabaaabaGaemOta4eaaOGaey41aqRaeeymaeJaeeimaaJaeeimaadaaa@3EFA@

Where *comp(i) *is the percent composition of a residue of type *i*. *Ri *and *N *are number of residues of type *i*, and total the number of residues in protein *i *(length of protein) respectively.

dpep(i)=DiN× 100
 MathType@MTEF@5@5@+=feaafiart1ev1aaatCvAUfKttLearuWrP9MDH5MBPbIqV92AaeXatLxBI9gBaebbnrfifHhDYfgasaacPC6xNi=xI8qiVKYPFjYdHaVhbbf9v8qqaqFr0xc9vqFj0dXdbba91qpepeI8k8fiI+fsY=rqGqVepae9pg0db9vqaiVgFr0xfr=xfr=xc9adbaqaaeGacaGaaiaabeqaaeqabiWaaaGcbaGaemizaqMaemiCaaNaemyzauMaemiCaaNaeiikaGIaemyAaKMaeiykaKIaeyypa0tcfa4aaSaaaeaacqWGebardaWgaaqaaiabdMgaPbqabaaabaGaemOta4eaaOGaey41aqRaeeiiaaIaeeymaeJaeeimaaJaeeimaadaaa@3F99@

Where dpep(i) is fraction or composition of dipeptide type i. Di and N are number of dipeptide of type i and number of overlapping peptides in protein i, respectively.

#### Evolutionary information

**Figure 3:**
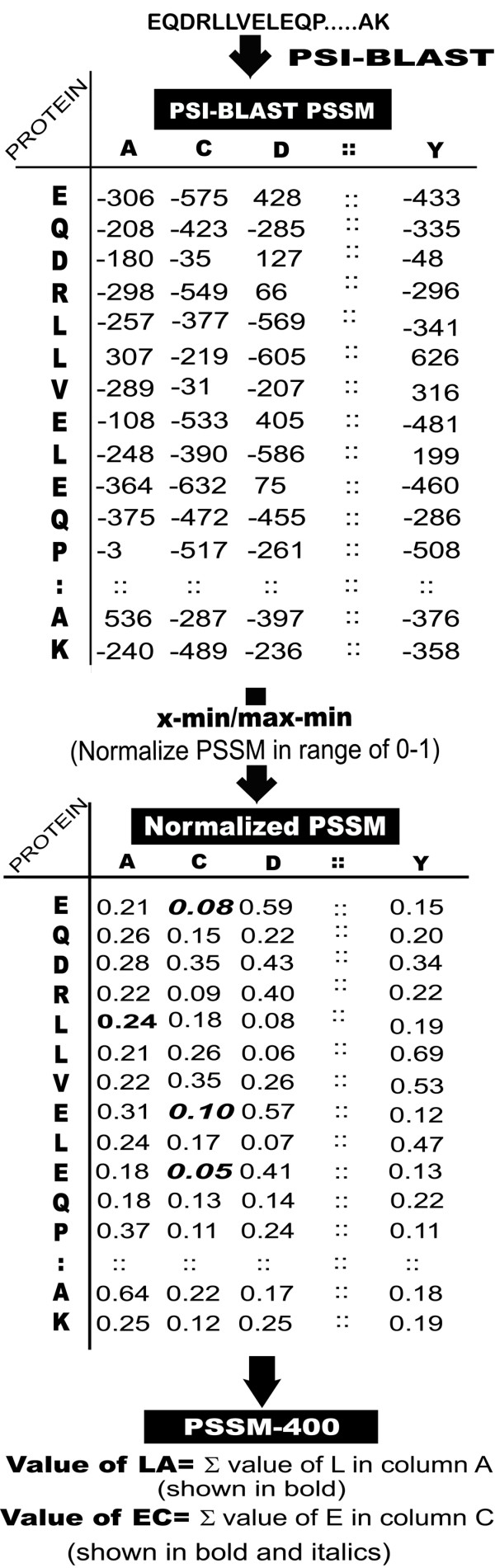
Schematic representation of algorithm used to convert 21*N dimensional PSSM into PSSM-400.

In past evolutionary information in form of position specific scoring matrix (PSSM) has been used for prediction of protein secondary structure [33-35]. Recently evolutionary information has been used for predicting subcellular localization of proteins [12,13]. In this study PSSM has been used for predicting DNA-binding proteins. The PSSM for each sequence was generated by PSI-BLAST search against 'nr' database using three iterations with e-value cut off 0.001. The PSSM contains probability of occurrence of each type of amino acid at each residue position of protein sequence. The evolutionary information in PSSM is presented by a matrix of dimension L × 21 (L rows and 21 columns) for a protein of length L where 21 columns represents occurrence/substitution of each type of 20 amino acids and dummy residue 'X' for insertion/deletion. We generated three vectors of dimension 21, 420 and 400, called PSSM-21, PSSM-420 and PSSM-400 respectively, from PSSM matrix. PSSM-21 is simple composition of occurrence of each type of amino acid, calculated by summing each column of PSSM. PSSM-420 is composition of occurrences of each type of amino acid corresponding to each type of amino acids in protein sequence; it means for each column we will have 20 values instead of one. Hence, we will have vector of dimension 20 × 21 for PSSM matrix. PSSM-400 is similar to PSSM-420 except dummy residue 'X' is ignored. It means dimension will be reduced to 20 × 20. We normalize the values of PSSM in range of 0–1 by using formula (Value-minimum)/(maximum-minimum) before computing vector PSSM-400 and PSSM-420. The process of converting L × 21 size matrix into PSSM-400 is diagrammatically shown in Figure 3. In this study we used mainly PSSM-400 (or PSSM) for developing models.

#### Four-part amino acid compositions

Protein sequence was divided into four non-overlapping equal length sub-sequences. Then amino acid composition of each sub-sequence was computed. Composition of each sub-sequence was concatenated together to make final input vector of dimension 80.

## Availability and Requirements

**Project name: **DNA-binding proteins prediction;

**Project home page: **;

**Operating system(s): **Platform independent;

**Programming language: **None;

**Licence: **No restriction;

**Any restriction to use by non-academics: **No restriction.

## Authors' contributions

MK carried out the data analysis and interpretation, developed computer programs, wrote the manuscript and developed the web server. MMG conceived the idea, created clean datasets and revised the manuscript critically for important intellectual as well as professional content. GPSR conceived and coordinated the project, guided its conception and design, helped in interpretation of data, refined the drafted manuscript and gave overall supervision to the project. All authors read and approved the final manuscript.

## Supplementary Material

Additional file 1 Contains 6 additional tables and 3 figures that can help in better understanding of the study described above. Click here for file
