# Supplementary material for: Identification of DNA-binding proteins using support vector machines and evolutionary profiles
Source: BMC Bioinformatics. 2007 Nov 27;8:463. doi: 10.1186/1471-2105-8-463 (PMC2216048; doi:10.1186/1471-2105-8-463)
Supplement: Additional file 1 — Contains 6 additional tables and 3 figures that can help in better understanding of the study described above. [file 1471-2105-8-463-S1.pdf]

Supplementary material

Identification of DNA-binding proteins using support vector machines and evolutionary profiles

Manish Kumar[1], M. Michael Gromiha[2] and G. P. S. Raghava[*1]

* Corresponding Author
[1]Bioinformatics Centre, Institute of Microbial Technology, Sector 39A, Chandigarh-160036, India

[2]Computational Biology Research Center, National Institute of Advanced Industrial Science and Technology, AIST Tokyo Waterfront Bio-IT Research Building, 2-42 Aomi, Koto-ku, Tokyo 135-0064, Japan.

Self-consistency Test

Self-consistency test is a method to evaluate the fitness of data in a prediction method. In self-consistency test, sequences of training sets will be predicted with decision rules derived from the same data. The accuracy of self-consistency reveals the fitting ability of the rules captured from the characteristics of training sets. Hence, it can be effectively used as an evaluation method to check the rigorousness and consistency of the prediction system. Since the prediction system parameters obtained by the self-consistency test are from the training dataset that includes the information of the query protein, error will be underestimated and the success rate is pretty high. However, it reflects the consistency of prediction method. We have trained the SVM on all 396 proteins of DNAset using PSSM-400 input. The SVM model generated by this training was used for prediction. Our method showed the specificity of 83.20% even at 100% sensitivity (Table S1). On the other hand at sensitivity of 75.34%, the specificity was 97.20%. The high sensitivity

and specificity of prediction clearly shows the robustness and consistency of prediction method.

Table S1: Self-consistency test of SVM model developed by using PSSM-400 of DNAset dataset as input.

Threshold	Sensitivity (%)	Specificity (%)	Accuracy (%)	MCC
-1.00	100.00	65.60	78.28	0.64
-0.90	100.00	74.40	83.84	0.72
-0.80	100.00	77.60	85.86	0.75
-0.70	100.00	80.00	87.37	0.77
-0.60	100.00	81.20	88.13	0.78
-0.50	100.00	81.60	88.38	0.79
-0.40	100.00	83.20	89.39	0.80
-0.30	99.32	84.00	89.65	0.81
-0.20	99.32	84.80	90.15	0.81
-0.10	98.63	86.40	90.91	0.82
0.00	98.63	87.60	91.67	0.84
0.10	98.63	89.20	92.68	0.86
0.20	97.26	91.60	93.69	0.87
0.30	97.26	94.40	95.45	0.91
0.40	95.89	95.20	95.45	0.90
0.50	94.52	95.60	95.20	0.90
0.60	93.15	95.60	94.70	0.89
0.70	92.47	96.40	94.95	0.89
0.80	91.10	96.40	94.44	0.88
0.90	87.67	96.80	93.43	0.86
1.00	75.34	97.20	89.14	0.77

Table S2: 5-fold cross-validation performance of PSSM based SVM model. (Learning parameter of SVM: j=2; t=1; d=1; c=0.0001). Here PSSM was generated in 5-fold cross-validation mode. During this procedure, whole dataset was randomly divided into five equal parts. Four sets were used as PSI-BLAST database (for PSSM generation) of remaining one set. This procedure was repeated five times so that each set was tested once. The performance shown here is average of all four sets.

Threshold	Sensitivity (%)	Specificity (%)	Accuracy (%)	MCC
-1.00	97.26	44.00	63.63	0.44
-0.90	96.60	46.40	64.90	0.45
-0.80	96.60	47.60	65.65	0.46
-0.70	95.91	50.80	67.42	0.48
-0.60	95.22	54.00	69.19	0.50
-0.50	94.55	56.40	70.46	0.51
-0.40	93.17	59.60	71.97	0.52
-0.30	91.82	61.60	72.73	0.52
-0.20	90.44	64.80	74.24	0.54
-0.10	88.39	68.00	75.51	0.54
0.00	86.32	70.80	76.52	0.55
0.10	83.56	74.00	77.53	0.56
0.20	80.83	77.60	78.79	0.57
0.30	78.76	80.00	79.54	0.58
0.40	73.93	82.40	79.29	0.56
0.50	70.53	83.60	78.78	0.54
0.60	66.41	86.00	78.79	0.54
0.70	63.01	89.60	79.80	0.56
0.80	57.49	90.80	78.53	0.53
0.90	52.03	92.80	77.77	0.51
1.00	46.58	94.00	76.52	0.48

Table S3: The performance of PSSM based SVM model developed on DNAset dataset and evaluated on independent dataset DNAiset (92 DNA-BPs and 100 NBPs).

	**Percent of correctly predicted**	
**Threshold**	**NBPs (Specificity)**	**DNA-BPs (Sensitivity)**
-1.00	70.00	86.96
-0.90	71.00	86.96
-0.80	75.00	83.70
-0.70	76.00	83.70
-0.60	76.00	82.61
-0.50	78.00	82.61
-0.40	79.00	81.52
-0.30	81.00	79.35
-0.20	84.00	78.26
-0.10	88.00	77.17
0.00	89.00	76.09
0.10	89.00	76.09
0.20	89.00	75.00
0.30	89.00	73.91
0.40	90.00	69.57
0.50	90.00	68.48
0.60	91.00	66.30
0.70	91.00	65.22
0.80	91.00	61.96
0.90	92.00	58.70
1.00	92.00	56.52

Table S4: Performance of similarity search methods on DNA-binding proteins of main (DNAset) dataset.

E-value	DNA-BLAST		DNA-PSI-BLAST	
	Total Hits	% Coverage	Total Hits	% Coverage
**0.001**	13 (12)	8.22	14 (13)	8.90
**0.01**	14 (13)	8.90	14 (13)	8.90
**0.1**	23 (15)	10.27	24 (15)	10.27
**1**	56 (22)	15.07	56 (22)	15.07
**10**	138 (40)	27.40	140 (42)	28.77

% Coverage indicates fraction of DNA-binding proteins, which showed DNA binding proteins at first hit from BLAST/PSI-BLAST search at a given threshold. Total hit is number of proteins, whose top-most hit has e-value less than the threshold. Values in parentheses show the number of correct hits from total hit.

Table S5: Performance of DBS-Pred on 100 DNA-binding and 100 non DNA-binding proteins.

Threshold (% Probability)	Correctly Predicted Binders (Sensitivity)	Correctly Predicted Non-binders (Specificity)	Overall Accuracy
0	100	0	50.00
10	83	31	57.00
20	77	46	61.50
30	69	57	63.00
40	56	63	59.50
50	49	75	62.00
60	39	82	60.50
70	31	85	58.00
80	26	87	56.50
90	17	92	54.50
100	0	100	50.00

Table S6. Performance of ANN using amino acid and dipeptide composition. Training was done for 20,000 cycles.

Input	Hidden Nodes	Step Size	Weight	Threshold	Sensitivity (%)	Specificity (%)	Accuracy (%)	MCC
**Amino acid composition**	11	0.1	0.02	0.2	67.86	68.80	68.46	0.36
**Dipeptide Composition**	5	0.1	0.02	0.2	62.32	60.00	60.84	0.22

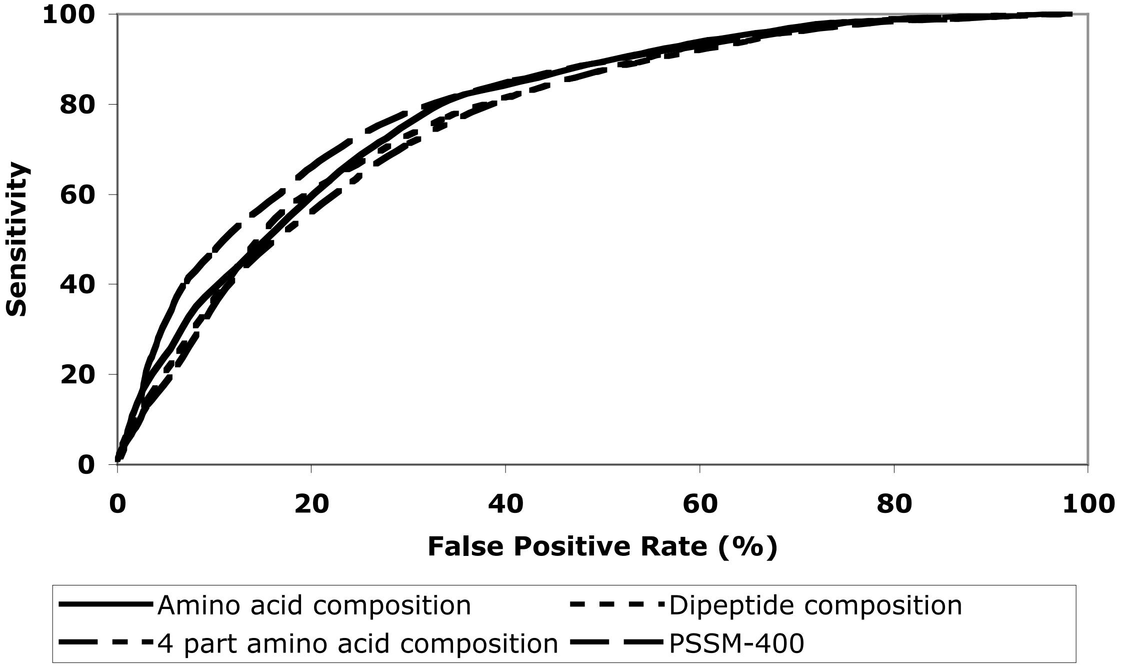

Figure S1: The performance of SVM on alternate dataset DNAaset (1153 DNA-binding and 1153 non-binding proteins) in the form of ROC plot.

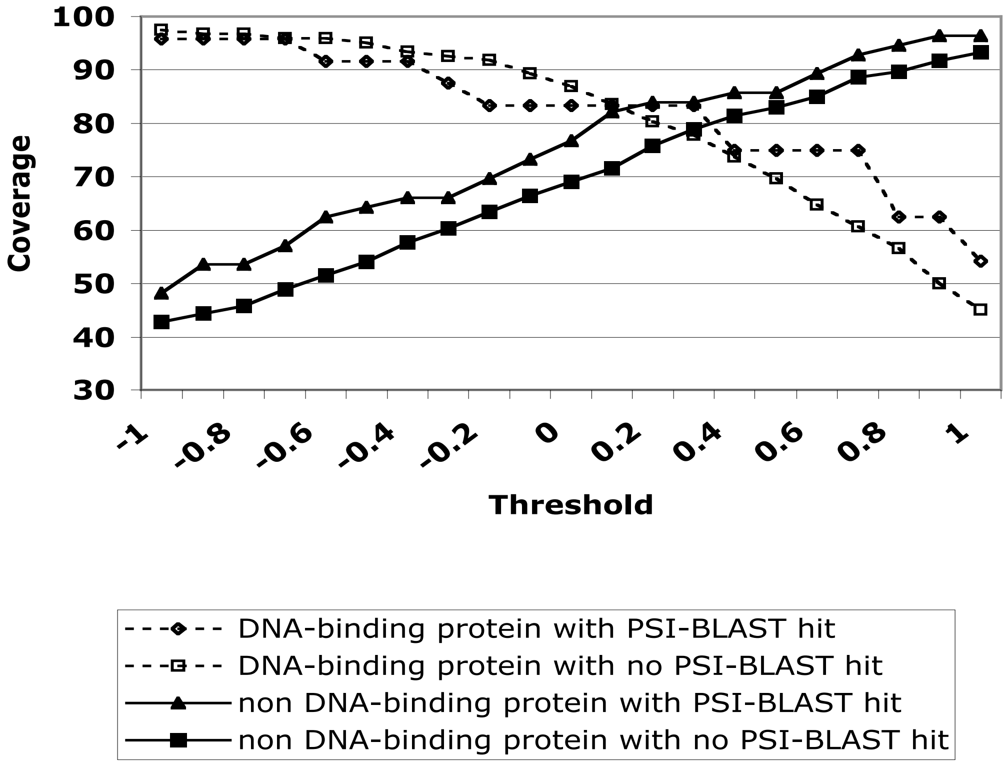

Figure S2: Effect of PSSM quality on performance of SVM.

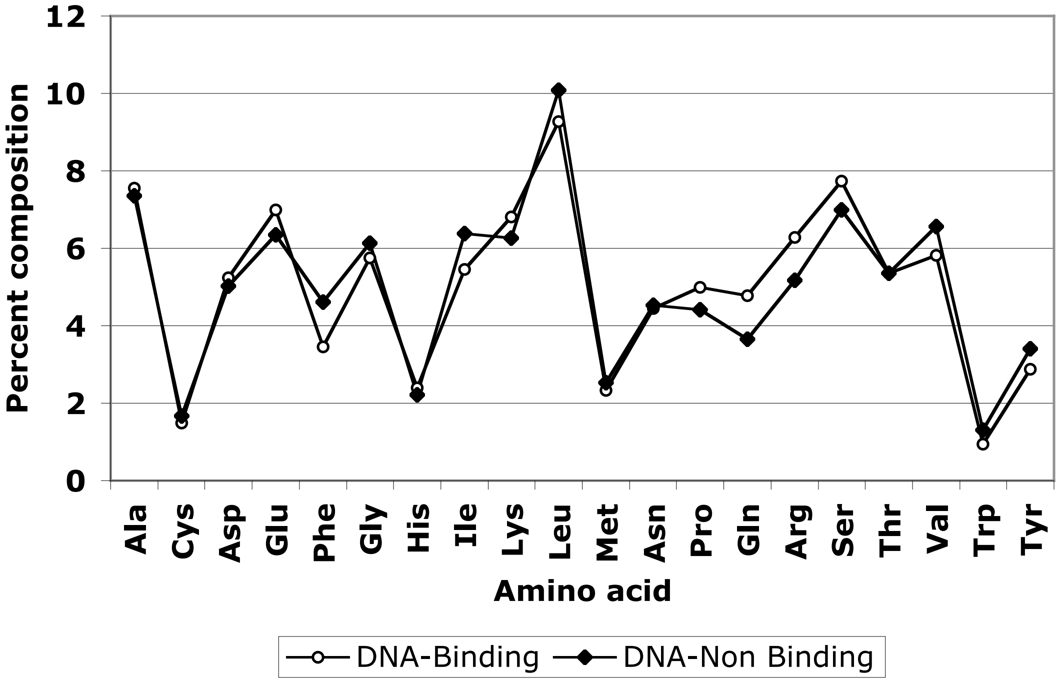

Figure S3: Percentage composition of amino acids in DNA-binding and non-binding proteins in DNAaset proteins**.**
